# Internet-Based HIV Prevention With At-Home Sexually Transmitted Infection Testing for Young Men Having Sex With Men: Study Protocol of a Randomized Controlled Trial of Keep It Up! 2.0

**DOI:** 10.2196/resprot.5740

**Published:** 2017-01-07

**Authors:** Brian Mustanski, Krystal Madkins, George J Greene, Jeffrey T Parsons, Brent A Johnson, Patrick Sullivan, Michael Bass, Rebekah Abel

**Affiliations:** ^1^ Department of Medical Social Sciences Feinberg School of Medicine Northwestern University Chicago, IL United States; ^2^ Institute for Sexual and Gender Minority Health and Wellbeing Northwestern University Chicago, IL United States; ^3^ Center for HIV Educational Studies and Training Hunter College, City University of New York New York, NY United States; ^4^ Graduate Center of the City University New York, NY United States; ^5^ Department of Biostatistics and Computational Biology University of Rochester Rochester, NY United States; ^6^ Department of Epidemiology Emory University Atlanta, GA United States

**Keywords:** eHealth, HIV prevention, Internet, risk reduction behavior, sexual behavior, sexually transmitted infections, young MSM

## Abstract

**Background:**

Human immunodeficiency virus (HIV) infections are increasing among young men who have sex with men (YMSM), yet few HIV prevention programs have studied this population. Keep It Up! (KIU!), an online HIV prevention program tailored to diverse YMSM, was developed to fill this gap. The KIU! 2.0 randomized controlled trial (RCT) was launched to establish intervention efficacy.

**Objective:**

The objective of the KIU! study is to advance scientific knowledge of technology-based behavioral HIV prevention, as well as improve public health by establishing the efficacy of an innovative electronic health (eHealth) prevention program for ethnically and racially diverse YMSM. The intervention is initiated upon receipt of a negative HIV test result, based on the theory that testing negative is a teachable moment for future prevention behaviors.

**Methods:**

This is a two-group, active-control RCT of the online KIU! intervention. The intervention condition includes modules that use videos, animation, games, and interactive exercises to address HIV knowledge, motivation for safer behaviors, self-efficacy, and behavioral skills. The control condition reflects HIV information that is readily available on many websites, with the aim to understand how the KIU! intervention improves upon information that is currently available online. Follow-up assessments are administered at 3, 6, and 12 months for each arm. Testing for urethral and rectal sexually transmitted infections (STIs) is completed at baseline and at 12-month follow-up for all participants, and at 3- and 6-month follow-ups for participants who test positive at baseline. The primary behavioral outcome is unprotected anal sex at all follow-up points, and the primary biomedical outcome is incident STIs at 12-month follow-up.

**Results:**

Consistent with study aims, the KIU! technology has been successfully integrated into a widely-used health technology platform. Baseline enrollment for the RCT was completed on December 30, 2015 (N=901), and assessment of intervention outcomes is ongoing at 3-, 6-, and 12-month time points. Upon collection of all data, and after the efficacy of the intervention has been evaluated, we will explore whether the KIU! intervention has differential efficacy across subgroups of YMSM based on ethnicity/race and relationship status.

**Conclusions:**

Our approach is innovative in linking an eHealth solution to HIV and STI home testing, as well as serving as a model for integrating scalable behavioral prevention into other biomedical prevention strategies.

**Trial Registration:**

Clinicaltrials.gov NCT01836445; https://clinicaltrials.gov/ct2/show/NCT01836445 (Archived by WebCite at http://www.webcitation.org/6myMFlxnC)

## Introduction

### Scientific Background

In the United States, young men who have sex with men (YMSM) are the group most affected by the human immunodeficiency virus (HIV) and acquired immune deficiency syndrome (AIDS) [1]. Over 70% of new HIV infections among youth and young adults occur in YMSM between the ages of 13 and 29 [2]. YMSM of color are disproportionately affected by HIV/AIDS, with African Americans and Latinos representing 45% and 28% of new HIV diagnoses, respectively, compared to whites who represent 16% of new HIV diagnoses [3]. The rate of new HIV infections also continues to increase among YMSM [2], making HIV prevention among this population a high priority research area [4,5].

Despite the burden of HIV among YMSM, few proven individual-level HIV prevention programs have been created specifically for this population [4]. Most of the evidence-based interventions (EBIs) recommended by the Centers for Disease Control and Prevention (CDC) focus on reducing HIV infections among heterosexual adults and other high risk youth [6,7]. Most EBIs designed for high risk youth do not specify a particular sexual orientation as an eligibility requirement or special topic of focus within the prevention program. Of the EBIs that are designed for men who have sex with men (MSM), few are designed specifically for younger men or ethnically and racially diverse MSM. One notable exception is the Young Men’s Health Project, which was evaluated with a diverse sample of YMSM (37% white, 29% Hispanic/Latino, 21% African American, and 13% other/multiple races) [8]. Participants completed four 1-hour motivational interviewing sessions delivered by therapists over a 12-week period.

To address the limited availability of EBIs for diverse YMSM, Keep It Up! (KIU!), an online HIV prevention intervention, was developed [9]. The intervention consists of interactive multimedia modules tailored to the real-life experiences of diverse YMSM. KIU! 1.0 was piloted in a randomized controlled trial (RCT) to test feasibility, acceptability, and preliminary efficacy [9]. In the pilot RCT, the intervention was delivered to YMSM upon receipt of a negative HIV test result from partner community-based organizations (CBOs). Participants in the intervention arm rated the program as valuable and acceptable, and reported statistically significant lower rates of condomless anal sex (CAS) at 3-month follow-up, compared to participants in the control arm [9]. Following the completion of the pilot RCT, the current multisite RCT (KIU! 2.0) was designed to test the efficacy of the intervention among a larger multisite sample of YMSM, with an extended follow-up assessment of behavioral and biomedical endpoints through 12 months. The multisite study design, online format, and yearlong follow-up period distinguish KIU! 2.0 from the Young Men’s Health Project that is currently available as an individual-level EBI for YMSM.

### Objectives

The overarching goal of the KIU! 2.0 project is to advance knowledge of technology-based behavioral HIV prevention, as well as improve public health by establishing the efficacy of an innovative electronic health (eHealth) prevention program for YMSM. We will accomplish these goals with three specific aims. First, we will integrate the KIU! intervention into a widely-used health technology platform to increase its scalability, adaptability, and potential for broad implementation. Second, we will test the efficacy of the KIU! intervention in a multisite RCT by (1) enrolling ethnically diverse HIV-negative YMSM (N=900; >65% ethnic/racial minorities) primarily in Atlanta, Chicago, and New York; (2) randomizing participants to either the KIU! intervention or an HIV knowledge control condition; and (3) measuring intervention outcomes at baseline and follow-up assessments at 3, 6, and 12 months.

The primary behavioral outcome will be the count of CAS acts, and the primary biomedical outcome will be incidences of sexually transmitted infections (STIs). Secondary behavioral outcomes include alcohol and drug use prior to sex, risky sex after substance use, condom errors, factors from the Information-Motivation-Behavioral Skills (IMB) theoretical model of HIV risk reduction [10], and receipt of an HIV test. We will test for dose effects based on metrics of intervention engagement and decay in intervention effects over time. Our third aim is to explore whether the KIU! intervention has differential efficacy based on the types of substances used prior to sex, as well as across important subgroups of YMSM based on race/ethnicity, gay/bisexual identity, and relationship status. In this context, *serious relationships* are defined as participants having a boyfriend/girlfriend or dating someone for an extended period of time and feeling very close to them, and *casual relationships* are defined as casual dating, sleeping with someone (eg, friends with benefits), having one night stands, or sex with strangers.

## Methods

### Trial Design

This is a two-group, active-control, double-blinded RCT of the online KIU! 2.0 intervention. Participants are randomized into two groups in equal proportions, and are blinded to which group is the intervention of interest. Consent materials indicate that we are evaluating two versions of an online HIV prevention program. Study investigators and staff who have contact with participants for enrollment and retention activities are also blinded to the arm in which participants are enrolled. The KIU! intervention includes seven modules that are completed across three sessions, at least 24 hours apart, totaling approximately 2 hours of content. Across these modules, the KIU! intervention uses diverse delivery methods (eg, videos, animation, and games) to address HIV knowledge, motivate safer behaviors, teach behavioral skills, and instill self-efficacy for preventive behaviors. The intervention is available on desktop, laptop, and tablet computers. Due to the Adobe Flash components of the intervention, KIU! 2.0 is not available on mobile devices. An earlier version of the intervention (KIU! 1.0) that did not contain the enhanced booster content at 3- and 6-month follow-ups, has been reported [9]. The control condition contains the same number of modules as the KIU! condition, with the same requirement to participate across three sessions. The control arm reflects HIV information that is currently available on many websites, with the aim to understand how the KIU! intervention improves upon information that is currently available online. Booster sessions are delivered at 3 and 6 months for each arm, and follow-up assessments are administered at 3, 6, and 12 months for each arm. Testing for urethral and rectal STIs is completed at baseline and 12-month follow-up for all participants, and at 3- and 6-month follow-up for participants who test positive for an STI at baseline. Participants are compensated in the following amounts: US $30 for baseline assessment and STI testing, US $20 for immediate posttest, US $20 for each 3- and 6-month follow-up assessment, and US $30 for 12-month follow-up assessment and STI testing. Some participants receive additional compensation. Participants who complete baseline assessment and STI testing at a university site or health department clinic receive an additional US $20, participants who complete STI testing at 3- and 6-month follow-up receive an additional US $10, and participants who are past due to complete their 12-month follow-up assessment and STI testing are incentivized with an additional US $20.

All procedures performed in this study are approved by the Emory University, Hunter College, and Northwestern University Institutional Review Boards. Informed consent is obtained from all individual participants included in this study.

### Participants

#### Eligibility Criteria

All interested participants are assessed for eligibility by completing a brief screener. Study inclusion criteria include (1) being between the ages of 18 and 29, (2) assigned male at birth and having current male gender identity, (3) receiving an HIV-negative test result from a study site or remote HIV testing, (4) reporting at least one act of CAS with a male partner in the prior 6 months, (5) not being in a behaviorally monogamous relationship lasting longer than 6 months, (6) having the ability to read English at an 8^th^grade level, and (7) having an email address that can be used for research contact for retention purposes.

#### Recruitment

**Figure 1:**
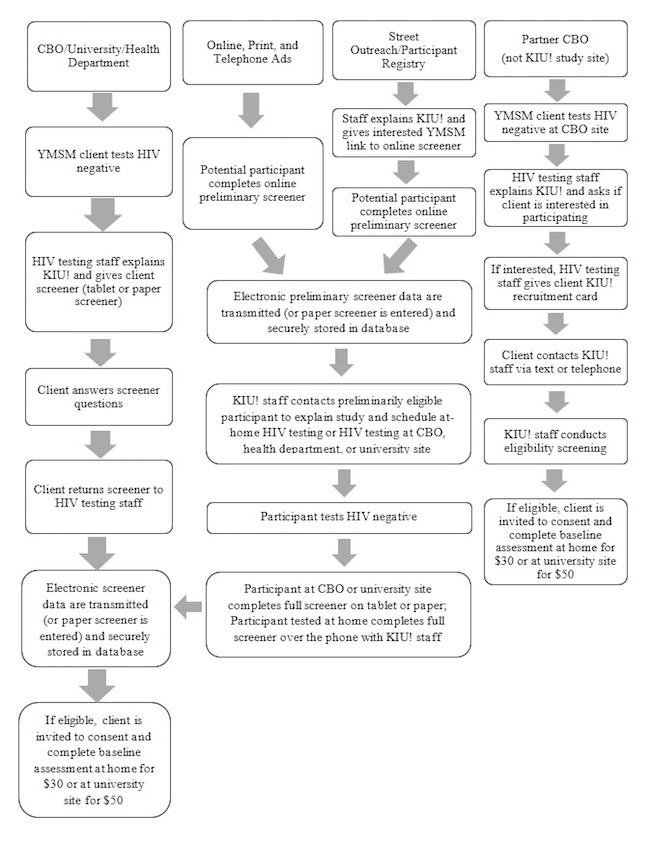
Recruitment strategies workflow, Keep It Up! 2.0.

Participants are recruited from a variety of sources including (1) HIV testing clinics and mobile testing units of our partner CBOs in Atlanta, Chicago, and New York; (2) university-based HIV testing at research sites in Atlanta and New York; (3) local health department clinics in Chicago; (4) street outreach by university staff in Atlanta, Chicago, and New York; (5) local and national print, online, and telephone-recorded ads; (6) referrals from completed observational studies and research participant registries at the university sites; (7) referrals from CBOs not affiliated with the study who provide HIV testing; and (8) nationwide online ads on social media apps linked with remote, at-home HIV testing (see Figure 1). Eligible participants across all study sites are offered the opportunity to visit Emory University, Hunter College, or Northwestern University to complete the baseline assessment and first set of self-administered STI test kits. Northwestern University and Emory University also offer Food and Drug Administration (FDA)-approved kits for at-home self-testing for HIV for participants recruited through these online and community-based recruitment methods.

#### Study Setting

Participants complete self-report assessments at baseline, immediately postintervention, and 3, 6, and 12-months postintervention. Assessments are completed via the Internet using a Computer-Assisted Self Interview. Participants are also mailed kits to collect urine and rectal swabs for STI testing at baseline and 12-month follow-up. Participants who test positive for an STI at baseline also provide samples for STI testing at the 3- and 6-month follow-ups, in addition to the 12-month follow-up.

### Intervention Arms

#### Keep It Up! Intervention Condition

In the context of a National Institutes of Health R34 grant, we collaborated with local CBOs to develop and pilot test KIU!, an interactive online HIV prevention project tailored to ethnically and racially diverse YMSM [9]. The intervention is informed by principles of e-learning [11] and based on the IMB model of HIV risk behavior change [10,12]. Our mixed-methods research, including qualitative interviews with ethnically-diverse YMSM, also directly informed the intervention design and content (eg, identification of myths about HIV transmission), particularly the refinement of messaging that was appealing across ethnic/racial groups [13].

The KIU! intervention includes seven modules completed across three sessions, completed at least 24 hours apart (ie, across at least 3 days), which total approximately 2 hours to complete. An innovative aspect of KIU! is that each module is based on a particular setting or situation that is relevant to the lives of YMSM, with developmentally appropriate health behavior change content embedded within each of these settings (see Table 1). For example, one intervention module follows a diverse cast of YMSM and highlights (1) the risks in making assumptions about a partner’s HIV status, (2) the risks of assumed monogamy in relationships, (3) the importance of regular HIV testing, (4) the skills for negotiating condom use within relationships, and (5) the limits of serosorting among HIV-negative YMSM. Across the KIU! modules, the intervention uses diverse delivery methods (eg, videos, animation, and games) to address gaps in HIV knowledge, motivate safer behaviors, teach behavioral skills, and instill self-efficacy for preventive behaviors. Additional information about the intervention content can be found in a previously published manuscript [9]. KIU! was piloted in an RCT to test feasibility, acceptability, and preliminary efficacy [9]. An ethnically and racially diverse sample of YMSM from the Chicagoland area was enrolled, excellent retention was achieved through 3-month follow-up (89%), and there was a significant 44% decrease in CAS relative to an active control group [9]. The intervention was then delivered as KIU! 1.5, a service project at a Chicago CBO from 2012 to 2014 [14].

**Table 1:** Intervention modules, Keep It Up! 2.0.

	Module	Style	Content
Session 1	Healthy and Whole Person	Diverse peer videos	The first module welcomes and engages participants in the KIU! intervention. Diverse YMSM are interviewed on the streets of Atlanta, Chicago, and New York and discuss connections to family, community, and romantic partners for setting positive norms for condom use and obtaining support from family of origin and choice [15,16].
	Hooking up Online	Stylized animation with three scenarios	This animated module follows three diverse YMSM chatting online with a focus on identifying triggers for CAS. Embedded content focuses on the effects of mood on risk [17,18], negotiating correct condom use, consequences of drug and alcohol on decision making [4], and facts about STI symptoms and prevention.
Session 2	The Club Game	Virtual reality game	In this interactive game, participants address pros/cons of condom use, steps to correct condom use, consequences of excessive alcohol consumption or drug use, issues with presuming HIV status in others, and effects of sexual arousal on decision making [4].
	Dating (an Older Partner)	Illustrated story in Flash animation	The power dynamics between an older and younger man in a dating relationship are explored as well as how YMSM can assert healthy behaviors [19]. Embedded in the module is a continuum of safer sex behaviors and strategies for implementing them.
Session 3	A Serious Relationship	Illustrated story in Flash animation and scripted scenarios on video	An illustrated story about dating considers ways to get sexual, emotional, and health needs met in relationships and how ongoing condom use can be an important aspect of that. The module also includes a video of a YMSM who receives an HIV-positive diagnosis while in a relationship. It wraps up with a video with actors portraying examples of good and bad communication about condom use.
	Setting Risk Reduction Goals	Health educator video and HIV prevention goals worksheet	Participants develop three realistic and practical goals based on topics covered in the intervention such as consistent condom use, regular HIV testing, and improving communication with partners. The purpose is to plan to engage in behaviors that preserve emotional, sexual, and physical health, and to troubleshoot obstacles to successful implementation of the goals.
	Sex in the City	Scripted soap opera -style video	A diverse cast of YMSM highlights the risks in making assumptions about a partner’s HIV status or monogamy, the limits of serosorting in HIV negative YMSM, the importance of regular testing, and skills for negotiating condom use within relationships. The soap opera is divided into four short videos that are shown across multiple sessions of the intervention. Part 1 is shown in the first session, part 2 in the second session, and parts 3 and 4 in the third session.
3 month follow-up	3 month booster	Scripted videos	A series of videos follow a young man named Antoine as he learns the importance of regular HIV testing and condom use after a condom failure due to incorrect use by his partner. Also included is video follow-up of a character from the “Sex in the City” soap opera who received an HIV negative test result and is working to maintain his risk reduction strategies. Participants are also given information about pre-exposure prophylaxis and other biomedical prevention strategies in various formats (video, fact sheet, and embedded Twitter feed). At the end of the booster, participants have a chance to revisit intervention modules and goals, troubleshoot obstacles to meeting goals, and set new goals or re-affirm existing ones.
6 month follow-up	6 month booster	Scripted videos	A series of videos follow Antoine as he navigates the dating scene before entering a serious relationship in which stopping condom use is discussed. In addition, participants have a chance to revisit 3 month booster content and goals, troubleshoot obstacles to meeting goals, and set new goals or re-affirm existing ones.

#### Control Condition

The control condition contains the same number of modules as the KIU! intervention condition, with the same requirement to participate across three sessions. Participants in the control condition also complete follow-up assessments and STI testing at the same time points as those in the KIU! condition. The control content reflects HIV information that is currently available on many websites, with the aim to understand how the KIU! intervention improves upon what is currently available online. Information on transmission, treatment, and prevention is provided through static slides with text and images. The control condition is didactic, not tailored to YMSM, noninteractive, and focuses on HIV/STI knowledge. Modifications were made to the control arm prior to the launch of KIU! 2.0 to include facts about biomedical prevention strategies. The use of this approach as a control condition ensures that both groups have equivalent access to the Internet for HIV-related content.

### Booster Sessions

In the current study, there are two booster sessions paired with follow-up assessments for both the intervention and control arms. These sessions occur at 3- and 6-month time points. At all follow-up time points, data collection occurs prior to booster session content, to prevent any effect on participant responses. The content provided at each follow-up varies by study arm.

#### Intervention Condition

The 3- and 6-month booster sessions reinforce learning from the intervention and provide additional HIV prevention information. The 3-month booster for the KIU! intervention focuses on the importance of repeat HIV testing, following the CDC’s recommendation of twice annual HIV testing among high-risk MSM [20]. The 6-month booster focuses on healthy romantic relationships, and is based on the findings that building and maintaining healthy relationships were two of the most popular topics in previous needs-assessments of online sexual health content for YMSM [21,22]. During each booster session, participants are provided the opportunity to review and update goals that they set during the postintervention assessment. At the end of each booster session, participants can review previous content. The 12-month follow-up assessment has no booster content and participants are administered the same measures that were previously completed at baseline and follow-up.

#### Control Condition

At the 3- and 6-month follow-up sessions, participants review content from the control modules. The slides on HIV information displayed in the initial modules are rearranged and administered at the 3-month follow-up. Slides with information on biomedical prevention strategies such as preexposure prophylaxis (PrEP), microbicides, and male circumcision are also included at the 3-month follow-up. The slides with STI information are rearranged and administered at the 6-month follow-up. Similar to the intervention condition, only the study measures are administered at the 12-month follow-up.

### Remote Testing for HIV and Sexually Transmitted Infections

#### HIV Testing

**Figure 2:**
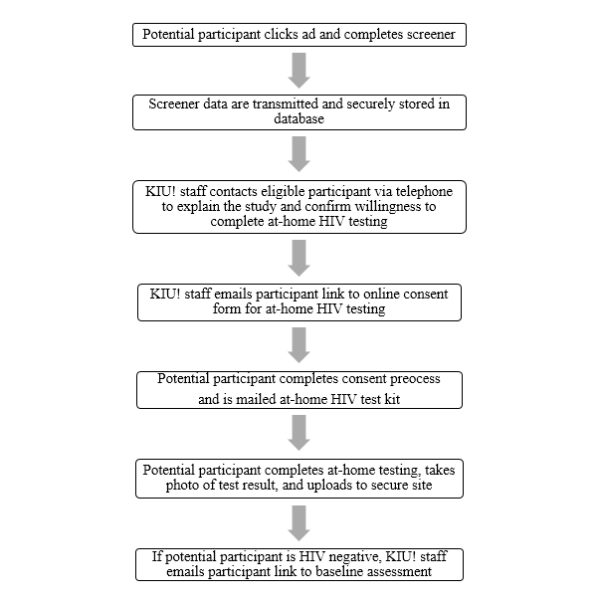
At-home HIV testing workflow, Keep It Up! 2.0.

To assess eligibility for participation in KIU! 2.0, individuals who are recruited online are mailed the FDA-approved, at-home, oral fluid OraQuick HIV test kit (see Figure 2). Following the instructions for self-testing that are included with the kit, participants self-administer the test and interpret their test result by comparing the test stick to the pictures and descriptions on the test kit directions. After determining their test result, participants report their result to study staff by uploading a photograph of the test stick to a secure online database. If the individual tests HIV-negative using the at-home test kit, the research assistant (RA) calls the participant to do a full screening over the telephone, and confirms eligibility. If eligible, an enrollment email with a link to the study is sent to the potential participant’s email address. If ineligible, the individual is informed that he is ineligible for KIU! but may be eligible for other studies. If he is interested in other studies, additional contact information is then collected. If the potential participant tests HIV-positive using the at-home test kit, the RA works with the individual to link them to care. The RA uses established organizational linkage-to-care procedures, including referring the individual to a clinic that will conduct a free confirmatory HIV test, and a referral to someone that will work with them to receive treatment if their confirmatory test returns positive. Research staff are responsible for reporting positive HIV test results to the appropriate health department.

#### Sexually Transmitted Infection Testing

To enroll in the study, potential participants are mailed at-home urine and rectal swab sample collection kits in a nondescript box to test for urethral and rectal gonorrhea (NG) and chlamydia (CT) at baseline. Easy-to-understand instructions for collecting and returning the samples are provided with the kits. In addition to the written instructions provided with the rectal STI kit, a video with instructions for properly collecting the rectal samples is shared with participants. The protocol for diagnostic testing of STI samples has changed as the study has progressed. Initially, the biotechnology company Identigene tested urine samples, while Emory University tested rectal swabs, for NG and CT. Both laboratories used the Nucleic Acid Amplification Test (NAAT) method, which is the gold standard method of diagnostic testing. As of March 2012, the CDC Division of Sexually Transmitted Diseases (STDs) Prevention laboratory provides diagnostic testing of the test kit samples using the NAAT method. Participants mail the kit to the CDC lab using prepaid boxes provided by the study. After STI test results are received by the KIU! 2.0 study team from the CDC, they are delivered to potential participants using a secure, encrypted email. To open the email and access their results, individuals must enter the unique study identification number that is provided to them with their test kit. Participants can print a hard copy of their results, and may speak to research staff if they wish. If positive STI test results arise, study staff provide local referrals for free or low-cost treatment and make a legally required confidential report to the appropriate health department. Across all stages of the study, the RAs prompt participants to access their results if they have not been viewed within 14 days of being made available. If a participant does not access his results after this reminder, the RA calls or sends additional reminders every 7 days. A minimum of three attempts at contact are made for both the reminders to return kits, and to access test results. If a participant does not respond to these attempts, the RA makes additional attempts for the duration of the study (as feasible) unless the participant explicitly asks to no longer be contacted. STI testing at follow-up follows the same protocol.

#### Integration with Patient-Facing Health Technology Platform

The online KIU! intervention was integrated into the online Web-based patient reported outcome (PRO) platform, Assessment Center (AC) [23,24]. The platform is a research management software application that serves as a library for PRO instruments, allows a mechanism for administering surveys, instruments, and forms to participants, and is a central facility for the storage, retrieval, organization, and sharing of study research items and data. The intervention integration consisted of adding a screening module that determined eligibility, and randomized participants into study arms. A tracking module was also developed and integrated with AC to assist in scheduling and managing the timely delivery of STI testing kits to study participants.

#### Participant Tracking and Retention

All participant tracking and retention activities are centrally managed at the lead site, Northwestern University, where the tracking technology is based. A supplemental database housed on REDCap, an online application, is used to log staff contact with participants and participant progress in the study. In consideration of difficult-to-reach participants across study sites, the Atlanta- and New York-based research staff members also assist with tracking and retention activities. The belief is that participants will be more responsive to contacts made from local sites, especially if they were recruited from these sites.

#### Randomization and Allocation

**Figure 3:**
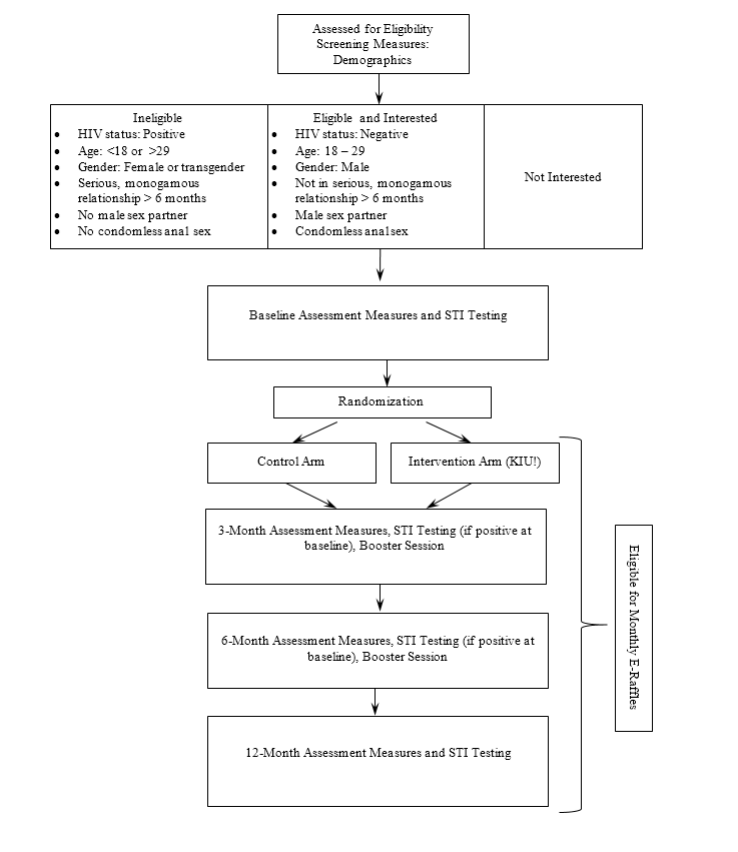
Participant workflow, Keep It Up! 2.0.

Upon enrollment, participants are randomly assigned by the online program (AC) to receive the KIU! intervention or HIV knowledge control arm. Participants do not know which group is the intervention under evaluation. Study investigators are blinded to the arm in which participants are enrolled. Randomization was performed using 6 permuted blocks of size 4, and stratified by race and HIV testing site at baseline [25]. Stratifying by race assures sufficient representations on each treatment arm to address the aim of exploring potential ethnic/racial differences in outcome effects. Stratification by HIV testing site prevents imbalance in latent geographical factors that may influence intervention responsiveness across cities and clinics. After the pretest assessment and remote STI testing, participants receive the intervention content across three sessions, over a minimum of 3 days and a maximum of 3 weeks, based on principles of effective HIV interventions and high acceptability in the pilot phase (KIU! 1.0). Participants maintain consistent online contact throughout the course of the study (a total of 12 months *after* intervention completion) and booster sessions and follow-up assessments are delivered at 3 and 6 months. The final follow-up assessment is administered at 12 months. All enrolled participants are also emailed a link to enter a monthly e-raffle for a US $50 gift card for the duration of their participation in the study. All participants who click the link and verify or update their contact information in an online survey are entered into the e-raffle. The monthly raffles are modeled on a previous study that used interim e-raffles to maintain participant engagement and up-to-date contact information [26]. The *Participant Flow Diagram* is presented in Figure 3.

## Results

A total of 2984 potential participants have been screened across all recruitment sources. Of those screened, approximately half were eligible, and 901 participants were enrolled to make up the final study sample (see Table 2). The sample is diverse with 36.6% (330/901) of participants identifying as non-Latino white. The mean age of the sample is approximately 24 years and most participants identify as gay, single, having at least some college education, and being employed at least part time. Close to half (408/901, 45.3%) of all participants identified as having no religious affiliation. Over 60% (560/901) of participants reported substance use in the past 3 months. Most participants (477/901, 52.9%) reported using marijuana, with poppers and cocaine being the second and third most commonly reported drugs (179/901, 19.9%; and 119/900, 13.2%, respectively). Approximately one third (252/841, 30.0%) of participants reported using substances in the four hours before having sex with their partners in the past 3 months.

**Table 2:** Demographic characteristics of enrolled Keep It Up! 2.0 participants.

Characteristics		n (%)
Total		901
**Race/Ethnicity**		
	White	330 (36.6)
	Latino	260 (28.9)
	Black	219 (24.3)
	Other	92 (10.2)
**Sexual identity**		
	Gay	777 (86.2)
	Bisexual	104 (11.5)
	Other	20 (2.2)
**Relationship status**		
	Serious relationship	175 (19.5)
	Casual dating	223 (24.8)
	Not in a relationship	501 (55.7)
**Religious affiliation**		
	Catholic	154 (17.1)
	Protestant	102 (11.3)
	No religious affiliation	408 (45.3)
	Other (eg, Jewish, Muslim)	237 (26.3)
**Education**		
	High school or less	113 (12.5)
	Some college	252 (28.0)
	College degree	418 (46.4)
	Graduate degree	118 (13.1)
**Current student**		
	Yes	328 (36.4)
	No	573 (63.6)
**Employment status**		
	Full time	451 (50.1)
	Part time	250 (27.8)
	Unemployed	199 (22.1)
**Substance use (past 3 months)**		
	Yes	560 (62.2)
	No	341 (37.8)
**Substance use before sex**		
	Yes	252 (30.0)
	No	589 (70.0)
Age, mean (SD)		24.3 (2.9)
Length (months) of serious relationship, mean (SD)		25.40 (28.6)

### Intervention Outcomes

Knowledge, motivation, skills (ie, partner sexual communication, correct condom use), and behavioral outcomes (ie, number of insertive and receptive CAS acts, condom errors) are measured at baseline and the 3-, 6-, and 12-month follow-up assessment time points. We measure intervention acceptability and tolerability immediately postintervention. Whenever possible, we selected measures designed for YMSM that were previously tested with diverse populations, to minimize cultural bias and maximize sensitivity and comparability to other studies. We follow participants for 12 months to assess behavioral outcomes far enough postintervention to allow for the potential occurrence of risk behaviors and HIV testing. This assessment plan also allows us to model possible degradation of treatment effects over time, and to assess outcomes 6 months after the final booster session, which meets CDC criteria for being classified as a tier I best-evidence HIV prevention program [6].

#### Primary Outcome Measures

The *HIV-Risk Assessment for Sexual Partnerships* (H-RASP) has been used with YMSM [27-29], and assesses sexual behaviors and associated situational and contextual variables on a partner-by-partner level, starting with recent partners, as well as in the aggregate. Partners are classified as serious or casual, and relationship duration is measured [19]. Questions differentiate between insertive and receptive anal sex. A sample question is, “How many times did you have sex without using a condom during anal sex (where you were the top) with this partner?”

The H-RASP measure includes a subset of questions specific to alcohol and drug use prior to sex, and is used to assess substance use as a risk factor for CAS. Substance use is being assessed as a risk factor because YMSM, in comparison to their heterosexual counterparts, are more likely to use a variety of different substances (including alcohol and illicit drugs), to initiate drug use at an earlier age, and to experience more rapid increases in substance use over time [30-33]. Substance use is also a primary risk factor for HIV in this population [4]. A sample question is, “How frequently did you use drugs in the 4 hours before having vaginal or anal sex with this partner?” Respondents indicate drug use via a 5-point frequency scale (1=never, 5=always) on a partner-by-partner level, as well as in the aggregate. For participants reporting drug use, a follow-up question assesses the particular drug(s) used.

To assess biomedical outcomes, urine and rectal samples are tested for NG and CT with the FDA-cleared Gen-Probe APTIMA Combo 2 Assay. All participants are tested at baseline and at the 12-month follow-up. Participants who test positive for an STI at baseline are also tested at the 3- and 6-month follow-ups. We test for both urethral and rectal NG and CT, as recent research shows rectal infections to be just as common, if not more so, than urethral infections, particularly among MSM of color [34]. In this study, the incidence of NG and CT serves as a biomedical endpoint for establishing intervention efficacy, and as a means for determining the feasibility of incorporating an innovative approach to STI testing into an online HIV prevention solution.

#### Secondary Outcome Measures

The *Brief HIV/AIDS Knowledge Questionnaire* is a true/false survey assessing knowledge of HIV transmission and prevention [35]. This questionnaire has strong internal consistency, test-retest stability [35], and has been used successfully with young adults [36]. Items are modified from the original measure to make them relevant for MSM. A sample question is, “Only the receptive/bottom partner is at risk of being infected with HIV during anal sex.” Correct answers are coded as 1 and incorrect or uncertain responses are coded as 0. Composite scores are calculated to reflect the percentage of correct responses.

The *HIV/AIDS Motivation and Behavioral Skills Questionnaire* [37] assesses motivation (eg, motivation to become safer), social norms (eg, partners’, friends’, or family members’ opinions about condom use), and behavioral skills (eg, negotiating condom use). Internal reliability Cronbach alphas range from .73 to .94 and the measure has been used and developed for MSM. A sample question is, “Based on your sexual behavior over the past 3 months, how much do you think you have been at risk for being infected with HIV or other STDs?”

The *Condom Errors Questionnaire* is an abbreviated version of the *Condom Use Errors and Problems Questionnaire* [38], which has been used with YMSM [39]. Using a 5-point Likert scale (1=never, 5=always) participants indicate the degree to which they had experienced a condom error (ie, using an oil-based lubricant), failure (ie, breakage during sex), or erection loss (ie, occurring prior to or during sex). A sample question is, “As a top during anal sex in the last 3 months, how often did you start having sex without a condom and then put it on later?”

The *Health Protective Communication Scale* [40] measures how respondents discuss health protection with their sex partners. This scale has been used with diverse adolescent and young adult samples (Cronbach alpha=.84 in a national sample) [40]. A sample item is, ‘‘How often in the past 6 weeks have you told a new sex partner that you would not have sex unless a condom is used?’’ Respondents rate items on a 4-point frequency scale (1=always, 4=never).

### Additional Measures

We use standard measures of age, ethnicity, education, and socioeconomic status. For YMSM, we use tailored items for gender identity, sexual orientation identity, and anatomic sex at birth.

The *PREP Intentions and Impact on Condom Use Measure* assesses participants’ intention to use PrEP, and is adapted from a measure used with high risk MSM [41,42]. A gateway question is used so that participants who have not used PrEP are asked about their intention to use it in the future, and participants who have used PrEP are asked about their use of, and attitudes towards, PrEP. Descriptive information on this measure in the KIU! 2.0 sample has been published [43].

The study team modified this measure at follow-up to better reflect PrEP use after it became FDA approved. For example, the baseline PrEP measure that was programmed before FDA approval of PrEP asks, “How many times have you taken anti-HIV medications?” under the assumption that participants might have been receiving PrEP inconsistently, as it was not readily available to most of the population. This question was removed in the follow-up assessments. New questions such as, “On a typical week, how many days did you miss taking your medication?” were added to the follow-up measure to reflect that participants who now take PrEP likely have a prescription for the medication, and to reflect the importance of assessing adherence.

The *Intervention Acceptability and Tolerability Measure* [44] includes a combination of open-ended questions (eg, “What aspect of the program did you like the least?”) and closed-ended Likert-style questions that form a scale of intervention acceptability (Cronbach alpha=.87). The questions were adapted from the original measure of 8 items to be specific to an online HIV intervention for adults. These adaptations were based on the investigators’ experience in the field, as were newly created items such as, “How interactive did you find the program?”

### Statistical Methods

Univariate summary statistics will be computed for all potential covariates. These summary statistics will be stratified by treatment arm, and then compared statistically through tests of two independent binomial proportions for binary variables, and two-sample t-tests for continuous variables to assess a failure of randomization. A Cochran-Mantel-Hanzel test of two independent binomial proportions will be used for the primary outcome measure of incident STIs at the 12-month endpoint, stratified by race and site, and an analogous stratified test for the count of CAS acts. These tests will set Cronbach alpha at .05, two-sided, and unadjusted for risk factors, except for the strata variables (race, site) used in the experimental design of the study. Ordinary generalized linear models and quasi-likelihood will be used to model the primary 12-month efficacy endpoints while adjusting for potential risk factors. Generalized linear mixed models and generalized estimating equations for multiple correlated, longitudinal CAS measures will be used to estimate the time-averaged treatment effect and time trends using all follow-up outcome measures, while adjusting for other potential time-dependent risk factors. The same regression modeling procedures will be used for secondary outcomes, such as condom errors, IMB factors, and receipt of an HIV test. All statistical analyses will be performed under an intent-to-treat principle [45].

To address potential adverse effects of participants’ use of PrEP during the study, we will use methods of causal inference under Rubin’s causal model [46,47] to adjust for postrandomization variables that allow for consistent estimates of treatment effects under the original study design, while adjusting for potential confounders. Principal stratification [48] will be used to conduct an analysis of the primary KIU! 12-month STI efficacy endpoint as well as CAS endpoint. Here, *any PrEP use* during the study is the principal strata, and this analysis is a comparison of two potential outcomes, had participants remained PrEP-free during the 12-month study. A complementary causal analysis is a regression model stratified by the propensity of PrEP use, where treatment arm is the primary covariate and the propensity score is constructed from a logistic regression model of PrEP use on potential confounders.

## Discussion

This evaluation of KIU!, a promising eHealth HIV prevention intervention for YMSM, is an important contribution to the field of HIV prevention for several reasons. To begin, while numerous funded studies regarding the Internet and HIV risk have been undertaken, there have been relatively few funded efficacy RCTs of HIV prevention eHealth projects, particularly among YMSM. Rates of HIV are on the rise among MSM in the period of *emerging adulthood*, but very little intervention research has been conducted with this high-risk group [4,28,49], therefore necessitating an efficacy trial among YMSM.

The KIU! intervention content and recruitment approaches also represent innovations in the field. Intervention content is based on the IMB theory of HIV risk behavior change [10,12,37,50], principals of e-learning [11], and qualitative research with ethnically and racially diverse YMSM, to ensure cultural relevance [13]. Content is delivered through videos, games, and animations to increase engagement and motivate behavior change by addressing peer norms, personal vulnerability, behavioral intentions, and examining safer sex practices (eg, the pros/cons of condom use). Significantly, KIU! uses a novel approach of focusing on situations (eg, dating an older partner) and settings (eg, Internet) commonly experienced by YMSM. Intervention content embedded within these *virtual* settings contrasts with traditional HIV prevention projects, which often have sessions focused on the standard topics of HIV knowledge, transmission, or prevention.

Regarding participant recruitment, KIU! is unique in linking a behavioral HIV prevention project to a clinical encounter (ie, HIV testing) as one of its recruitment strategies. Currently, most testing clinics have limited time and resources to provide prevention resources. This approach produces innovative research on how to catalyze prevention by capitalizing on a key clinical encounter that could then be generalized to other biomedical strategies that require embedded behavioral prevention (eg, PrEP). Such an intervention could play an important role in providing accessible prevention for YMSM, particularly YMSM seeking HIV testing. This approach represents an opportunity to develop a cost-effective and easy-to-use intervention that will engage and motivate participants, while teaching risk reduction behaviors. Additionally, recruitment may be extended beyond the clinic setting to include more traditional recruitment efforts, such as community and street outreach and organization referrals, as well as increasingly common online advertising. As demonstrated in this study, recruitment of diverse YMSM from a variety of sources is feasible for online HIV prevention research. Documenting these efforts will produce research on differences in retention and risk profiles of YMSM recruited from a variety of sources.

Another important contribution to the field of HIV prevention is our approach to incorporate STI testing into an online HIV prevention project, primarily through remote self-testing. This approach is in response to calls to incorporate STI testing and treatment into HIV prevention efforts [51-53], given that STIs are important risk factors in HIV transmission and acquisition due to increased biological susceptibility [54,55]. Additionally, in efficacy trials of sexual risk reduction interventions, STI infections can serve as sensitive biomarkers, particularly when HIV infection rates are too low to allow sufficient power with attainable sample sizes [56-60]. In KIU! 2.0, a portion of individuals were recruited from nationwide online ads; however, to assess eligibility, these potential participants were required to complete remote, at-home HIV testing. Upon successful enrollment into the study, all participants also completed STI testing as a means of generating a biological study endpoint for establishing intervention efficacy. Together, these strategies represent a public health solution for incorporating HIV and STI testing into an eHealth HIV intervention. To our knowledge, KIU! 2.0 is the first intervention to link remote STI testing into an eHealth HIV prevention intervention.

### Limitations

There are important limitations in considering the promise of KIU! 2.0 in its current form. The first limitation concerns access to the Internet for the delivery of online interventions. The Internet has become an important delivery approach for eHealth tools. Online interventions can be convenient for users as they are accessible from anywhere that there is a connection to the Internet. Additionally, online interventions can be used in private settings, which also improves accessibility and engagement without the fear of stigma, particularly for YMSM and other high-risk populations. Although the digital divide is narrowing, the promise of eHealth interventions may be limited for those without consistent and reliable Internet access. Second, issues related to the technology required to deliver and maintain eHealth interventions may serve as a study limitation. Currently, KIU! can only be accessed on laptops or computer tablets because content is not formatted for access on mobile phones. This factor may limit intervention access, particularly among subpopulations who primarily access the Internet via smartphones. Additionally, as with all Web-based applications, regular maintenance is required to ensure that the intervention is compatible with new and updated Web browsers, and to fix emerging bugs that impede participants’ ability to complete intervention sessions. Technical support and ongoing maintenance will present a financial challenge to future implementation after this trial is completed. Third, there is the challenge of deciding when and if to update eHealth intervention content during an ongoing RCT as new advances emerge (eg, PrEP) [61]. For example, PrEP became FDA-approved after the trial began, and therefore information on PrEP was added to booster sessions to assure all participants had access to this new information. Despite these considerations, computer- and Internet-based HIV prevention efforts show promise [62]. These resources have the advantages of standardization and ease of replication, as well as the added benefit of reach and increased use (particularly among youth), and are important venues for health interventions [63].

### Conclusions

The overarching goal of KIU! 2.0 is to advance scientific knowledge of Internet-based behavioral HIV prevention, and improve public health by establishing the efficacy of an innovative eHealth prevention program for YMSM. This research is making significant progress towards achieving the specified aims. First, the KIU! technology has been successfully integrated into a widely-used health technology platform to increase its scalability, adaptability, and potential for broad implementation. Second, baseline enrollment for the RCT is complete (N=901) and we are currently assessing intervention outcomes (ie, count of CAS acts and STI incidence) via follow-up assessments at 3, 6, and 12 months. Finally, upon collection of all data, and after the efficacy of the intervention has been evaluated, we will explore whether the KIU! intervention has differential efficacy across subgroups of YMSM based on ethnicity/race, relationship status, and other variables. Our approach is innovative in linking an eHealth solution to HIV and STI testing, and serves as a model for integrating scalable behavioral prevention into other biomedical prevention strategies.

### Trial Status

Participant recruitment for KIU! 2.0 is complete. Follow-up data is currently being collected and will be completed in early 2017.

## Abbreviations

AC: Assessment Center
AIDS: acquired immune deficiency syndrome
CAS: condomless anal sex
CBO: community-based organization
CDC: Centers for Disease Control and Prevention
CT: chlamydia
EBI: evidence-based intervention
eHealth: electronic health
FDA: Food and Drug Administration
HIV: human immunodeficiency virus
H-RASP: HIV-Risk Assessment for Sexual Partnerships
IMB: Information-Motivation-Behavioral Skills
KIU!: Keep It Up!
MSM: men who have sex with men
NAAT: Nucleic Acid Amplification Test
NG: gonorrhea
PrEP: preexposure prophylaxis
PRO: patient reported outcome
RA: research assistant
RCT: randomized controlled trial
STD: sexually transmitted disease
STI: sexually transmitted infection
YMSM: young men who have sex with men
